# Supplementary figures and images for: Red-light is an environmental effector for mutualism between begomovirus and its vector whitefly
Source: PLoS Pathog. 2021 Jan 11;17(1):e1008770. doi: 10.1371/journal.ppat.1008770 (PMC7822537; doi:10.1371/journal.ppat.1008770)

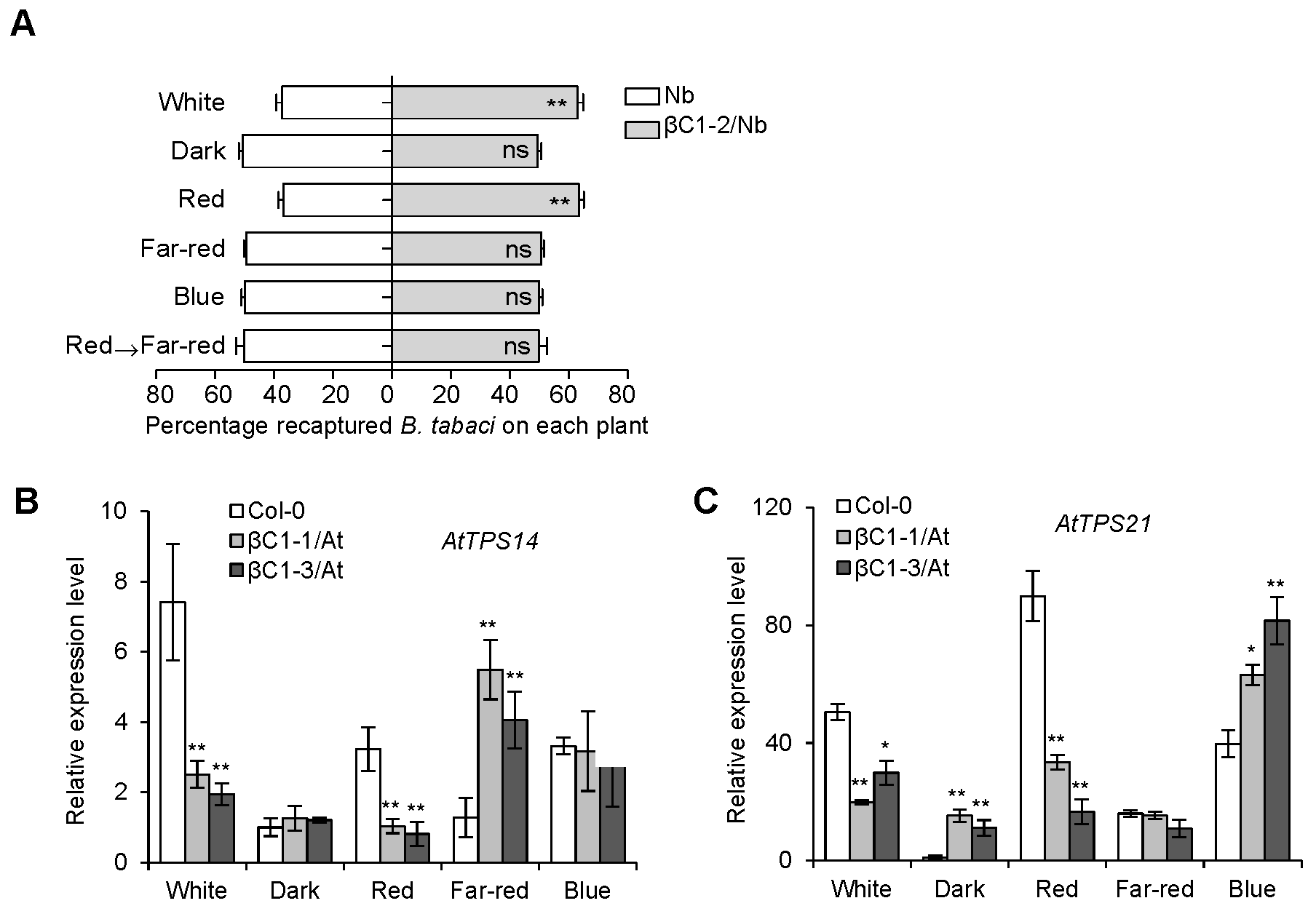

Supplement: S1 Fig — (A) Whitefly preference on wild-type Nb and βC1 transgenic Nb plants (βC1-2/Nb) in response to white, dark, red, far-red, and blue light. Plants were placed under darkness for 24 h, followed by a 2 h light exposure and then performed whitefly choice experiments. Red→Far-red indicates that plants were firstly kept in darkness for 24 h, followed by a 2 h red light exposure, and then transferred to far-red light for 2 h. Values are mean + SD (n = 6) (**, P< 0.01; ns, no significant differences; the Wilcoxon matched pairs test). (B-C) Relative expression levels of AtTPS14 (B), and AtTPS21 (C) in Col-0 and two βC1/At plants (βC1-1/At and βC1-3/At) under different light conditions. Values are mean ± SD (n = 3) (*, P< 0.05; **, P< 0.01; Student’s t-test). The light was supplied by LED light sources, with irradiance fluency rates of: white (80 μmol m-2 sec-1), blue (15 μmol m-2 sec-1), red (20 μmol m-2 sec-1), and far-red (2 μmol m-2 sec-1). (TIF) [file ppat.1008770.s001.tif]

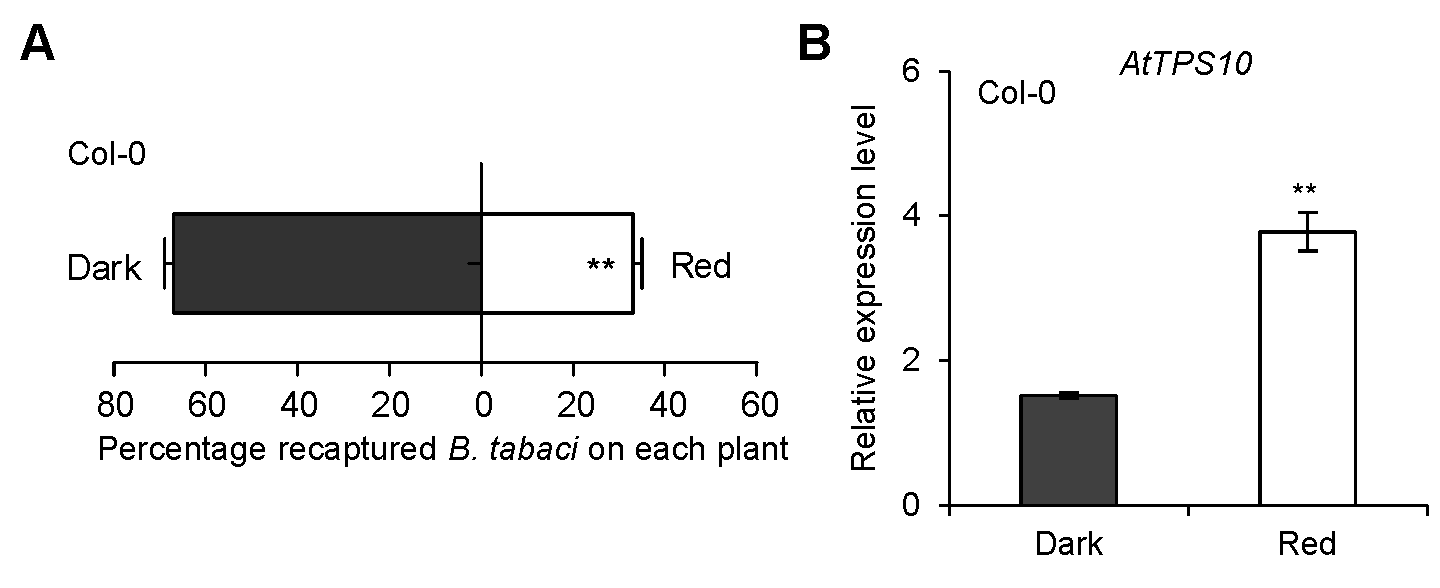

Supplement: S2 Fig — (A) Whitefly preference (as percentage recaptured whiteflies out of 200 released) on wild-type Col-0 in response to darkness or red light. The plants were placed in darkness for 24 h prior to the 2 h dark or 2 h red light (20 μmol m-2 sec-1) treatments. Values are mean + SD (n = 6) (**, P< 0.01; the Wilcoxon matched pairs test). (B) Relative expression levels of AtTPS10 in Col-0 plants exposed to darkness or red light. Values are mean ± SD (n = 3). Asterisks indicate significant differences of AtTPS10 expression in Col-0 plants between under darkness and red light (**, P< 0.01; Student’s t-test). (TIF) [file ppat.1008770.s002.tif]

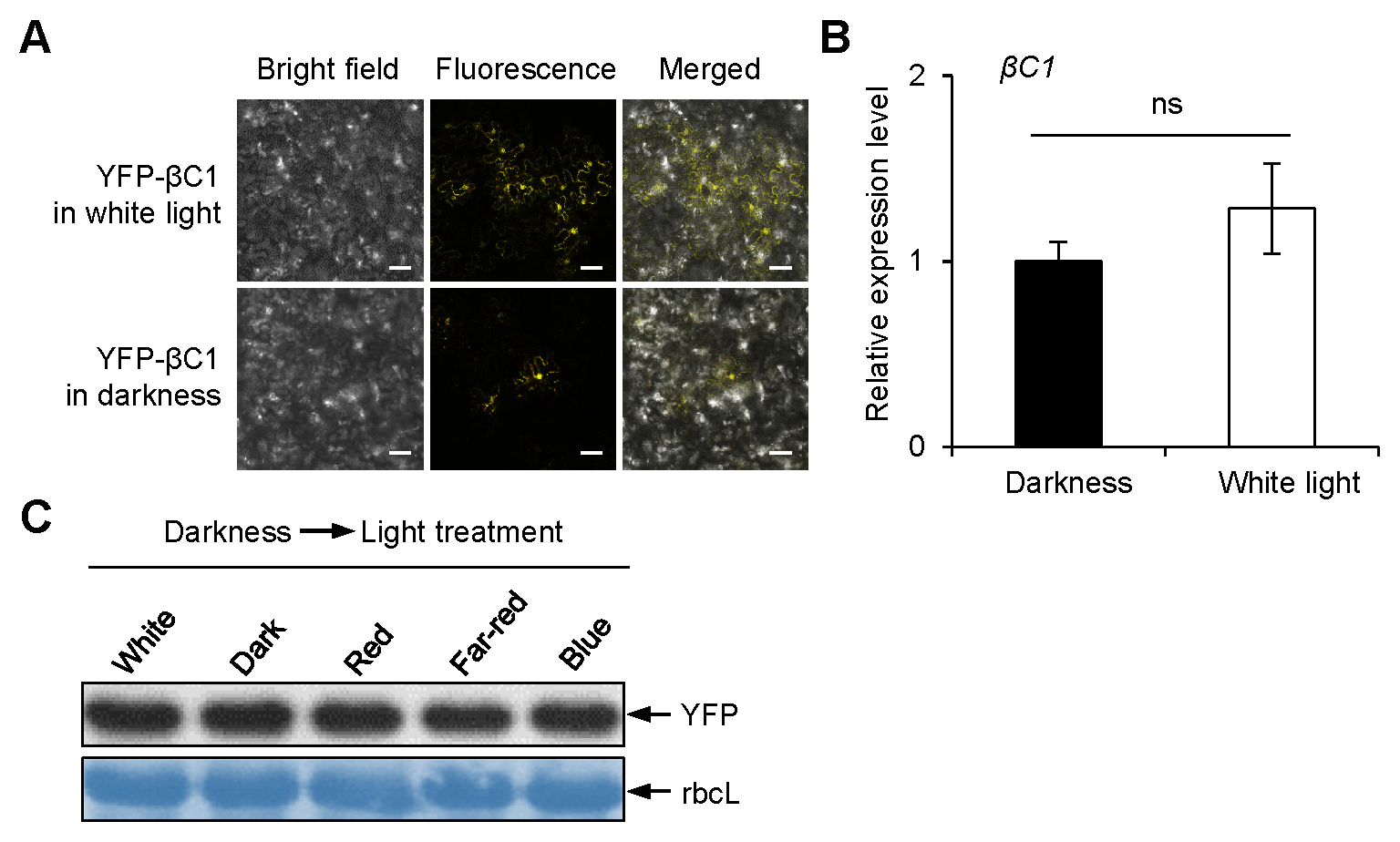

Supplement: S3 Fig — (A) Subcellular localization of YFP-βC1 in N. benthamiana under darkness or white light condition. After transient inoculation of 35S:YFP-βC1, plants were placed in the dark or in the white light for 48 h prior to the observation. Scale bars = 50 μm. (B) Relative expression levels of βC1 in Col-0 plants in response to dark or white light. Values are means ± SD (n = 3). ‘ns’ indicates no significant differences. (C) Accumulation of YFP proteins in Nb plants after different light treatments for 2h. Plants were agroinfiltrated with 35S:YFP, incubated in the dark for 60 h, and followed by a 2 h light exposure. Stained membrane bands of the large subunit of Rubisco (rbcL) were used as a loading control. (TIF) [file ppat.1008770.s003.tif]

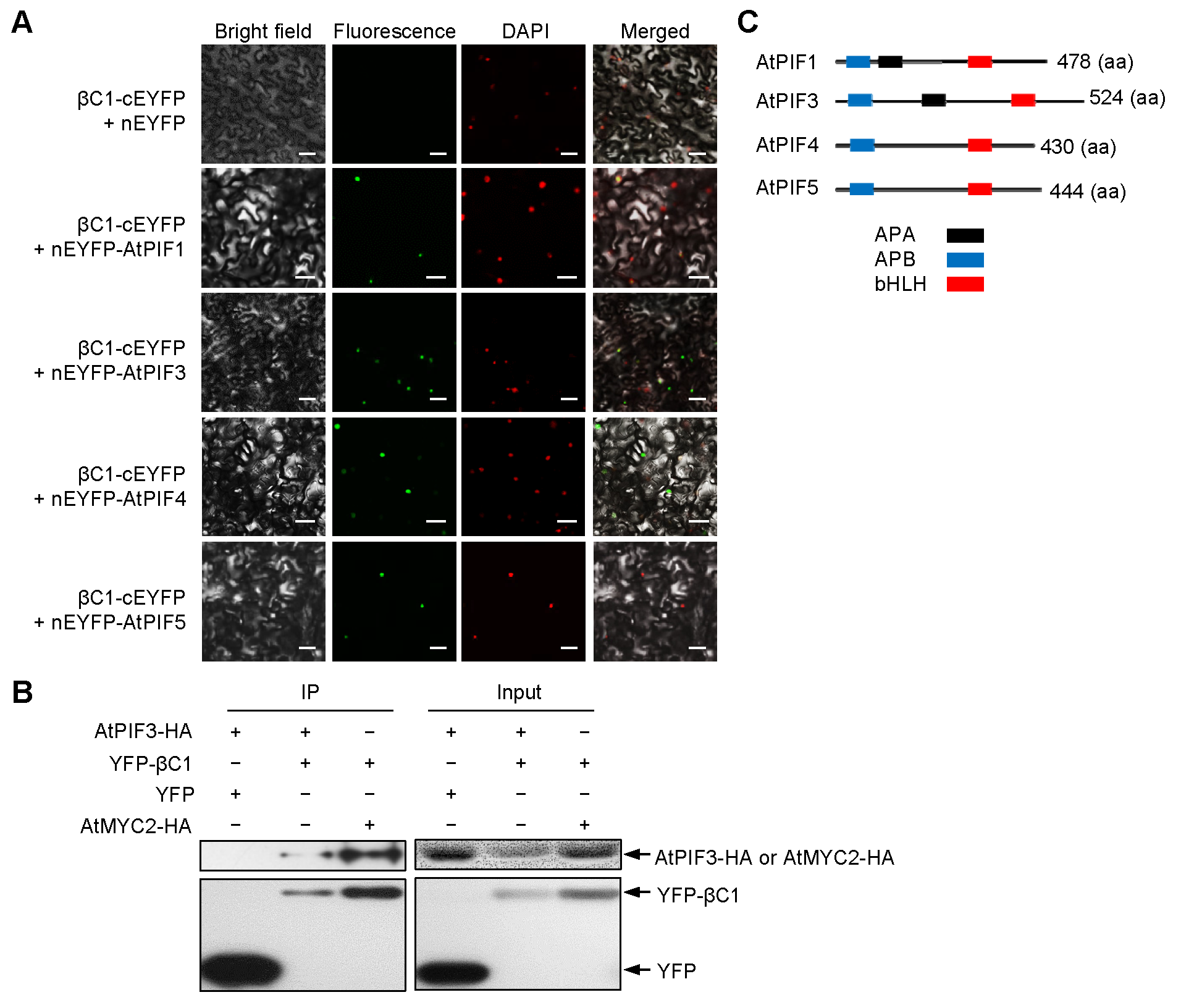

Supplement: S4 Fig — (A) Co-IP analysis of AtPIF3-HA and YFP-βC1 interaction in vivo. YFP was used as a negative control, while AtMYC2-HA was used as a positive control. All of above interaction experiments were performed in normal light condition. (B) In vivo BiFC analysis of βC1 interaction with Arabidopsis PIFs (AtPIF1, AtPIF3, AtPIF4 or AtPIF5). Fluorescence was observed owing to complementation of the βC1-cEYFP fused protein and nEYFP-AtPIFs fused protein. Nuclei of Nb leaf epidermal cells were stained with DAPI. Unfused nEYFP was used as a negative control. Scale bars = 50 μm. (C) Domain structure of AtPIFs proteins. Schematic diagrams of the AtPIFs polypeptide show the location of the consensus basic helix-loop-helix (bHLH) domain, which defines this transcription factor family, as well as the Active Phytochrome A-binding (APA) region and the Active Phytochrome B-binding (APB) region. (TIF) [file ppat.1008770.s004.tif]

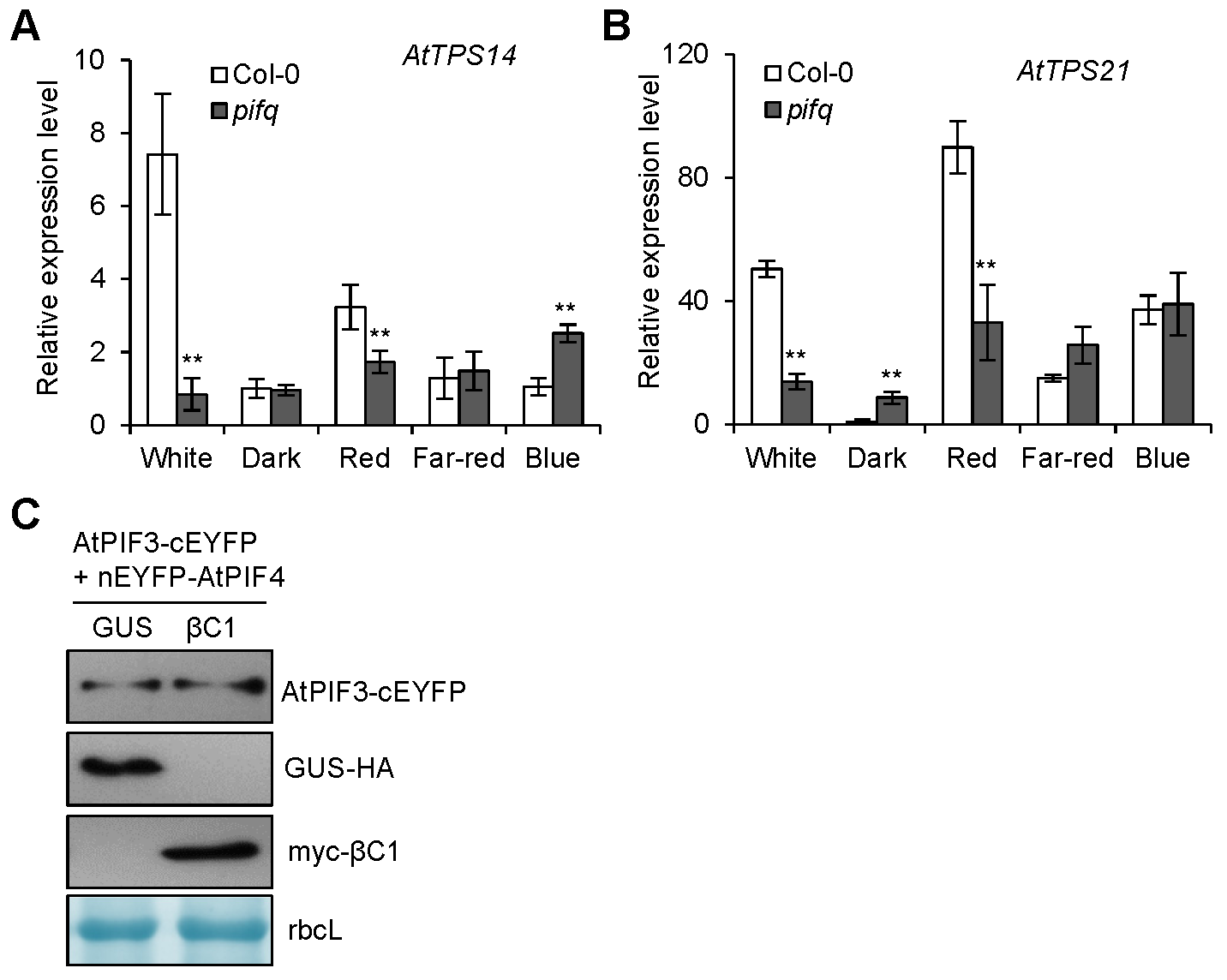

Supplement: S5 Fig — (A-B) Relative expression levels of AtTPS14 (A) and AtTPS21 (B) in Col-0 and pifq mutant plants after a 2 h treatment of different lights. Values are mean ± SD (n = 3) (**, P< 0.01; Student’s t-test). (C) Detection of protein levels in modified BiFC assay of Fig 5A. (TIF) [file ppat.1008770.s005.tif]

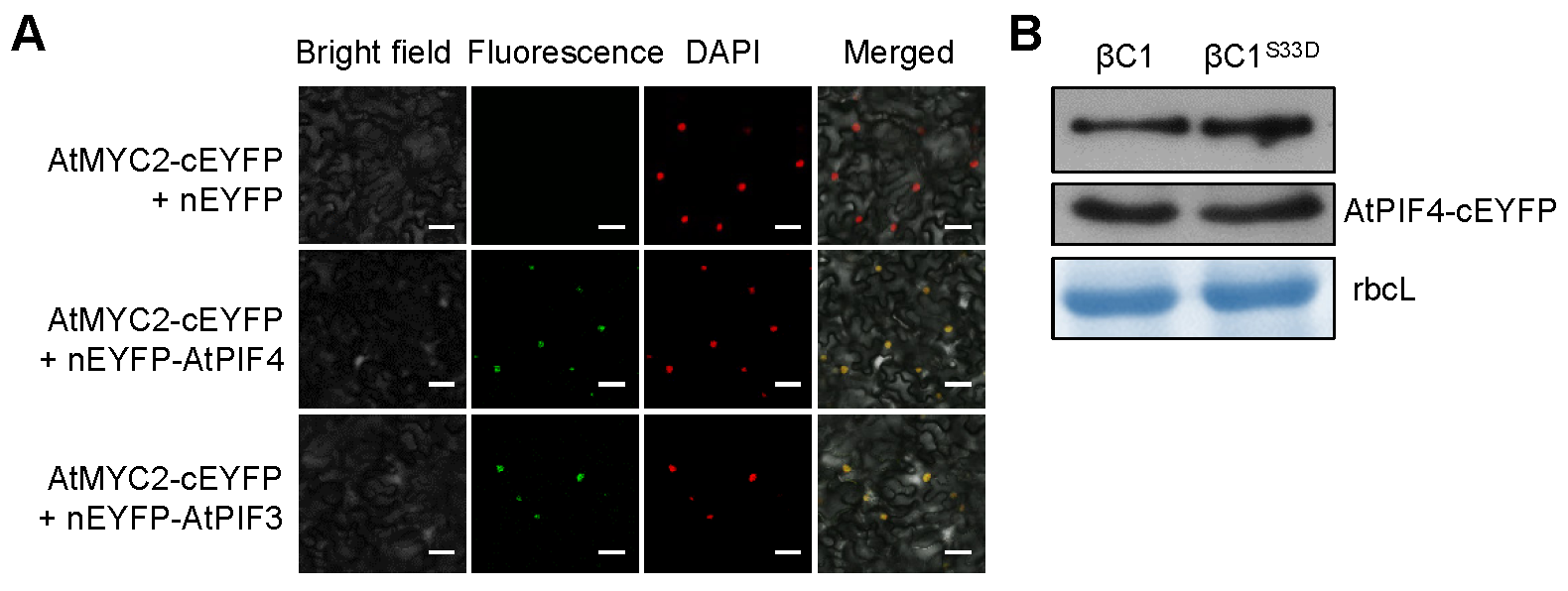

Supplement: S6 Fig — (A) In vivo BiFC analysis of AtMYC2 interaction with AtPIFs (AtPIF3 or AtPIF4) in normal light. Scale bars = 50 μm. (B) Detection of protein levels in modified BiFC assay of Fig 7C. (TIF) [file ppat.1008770.s006.tif]

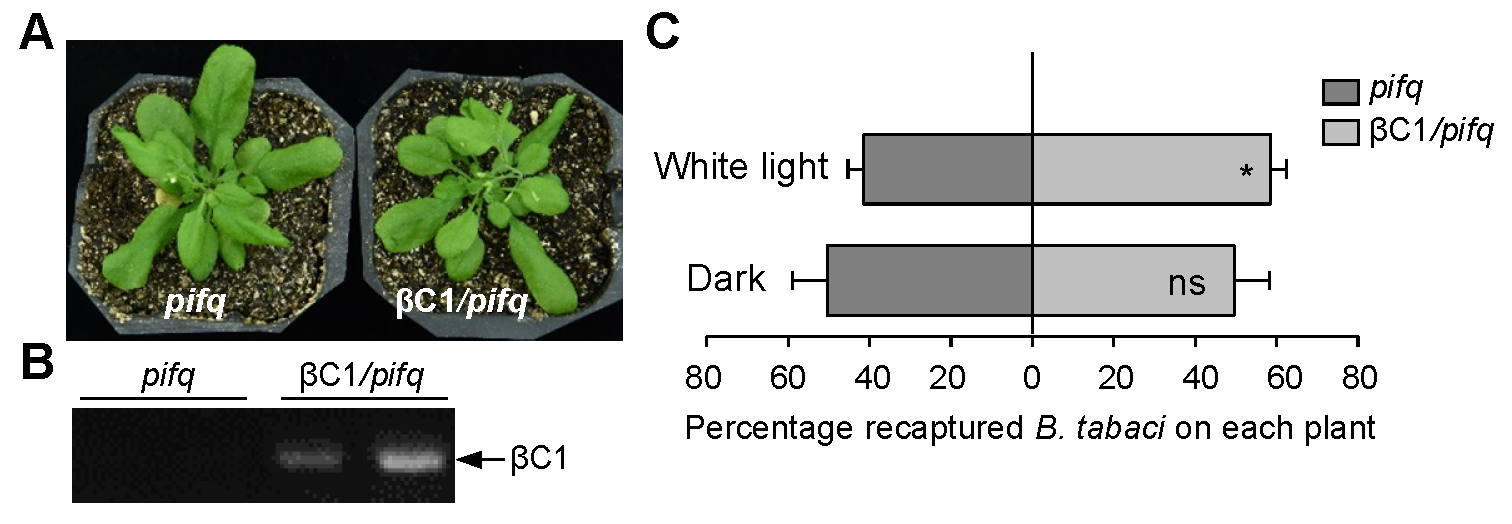

Supplement: S7 Fig — (A) The phenotype of the additional expression of βC1 in pifq mutant (βC1/pifq mutant). (B) Detection of βC1 expression in the βC1/pifq mutant by semi-quantitative PCR. (C) Whitefly preference on pifq mutant and βC1/pifq mutant in response to white light and dark. Values are mean + SD (n = 6) (*, P< 0.05; ns, no significant differences; the Wilcoxon matched pairs test). (TIF) [file ppat.1008770.s007.tif]

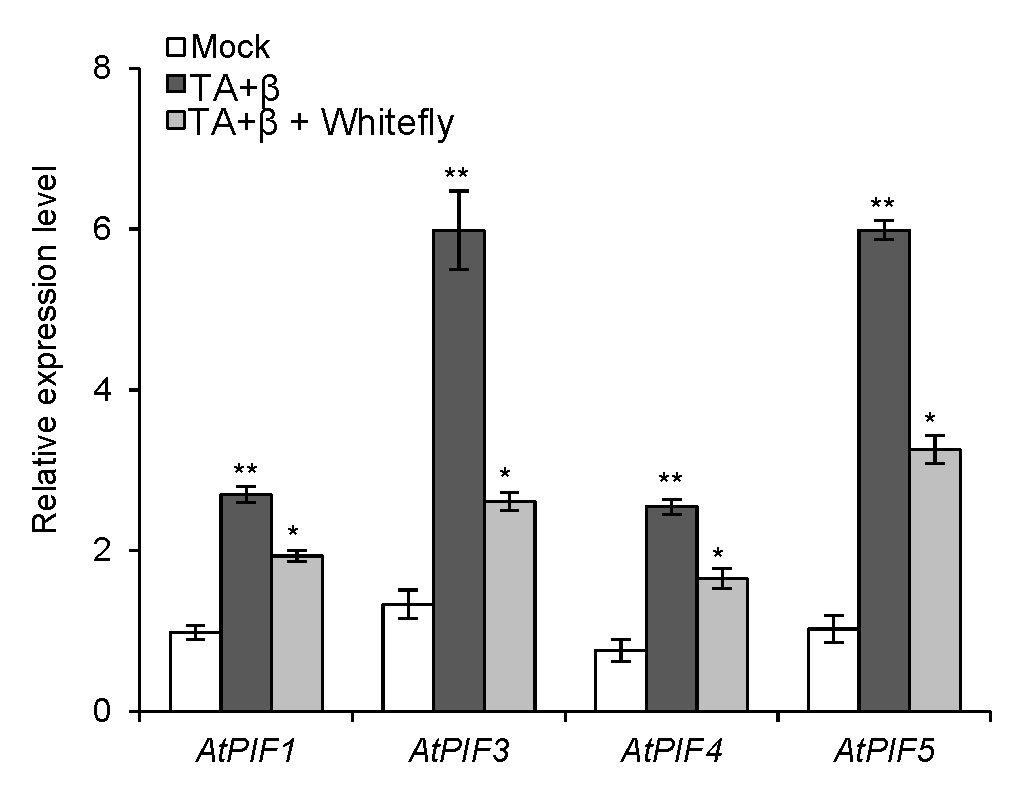

Supplement: S8 Fig — Relative expression levels of AtPIFs in Arabidopsis plants. Arabidopsis Col-0 plants agroinfiltrated with the infectious clones of TA+β complex at 14 dpi, followed by infestation by whiteflies for 6 h. Total plant RNAs were extracted for qRT-PCR analysis. Uninfected Col-0 plants were used as mock. Values are means ± SD (n = 3). Asterisks indicate significant differences of AtPIF genes expression between mock and infected-Col-0 plants (*, P< 0.05; **, P< 0.01; Student’s t-test). (TIF) [file ppat.1008770.s008.tif]

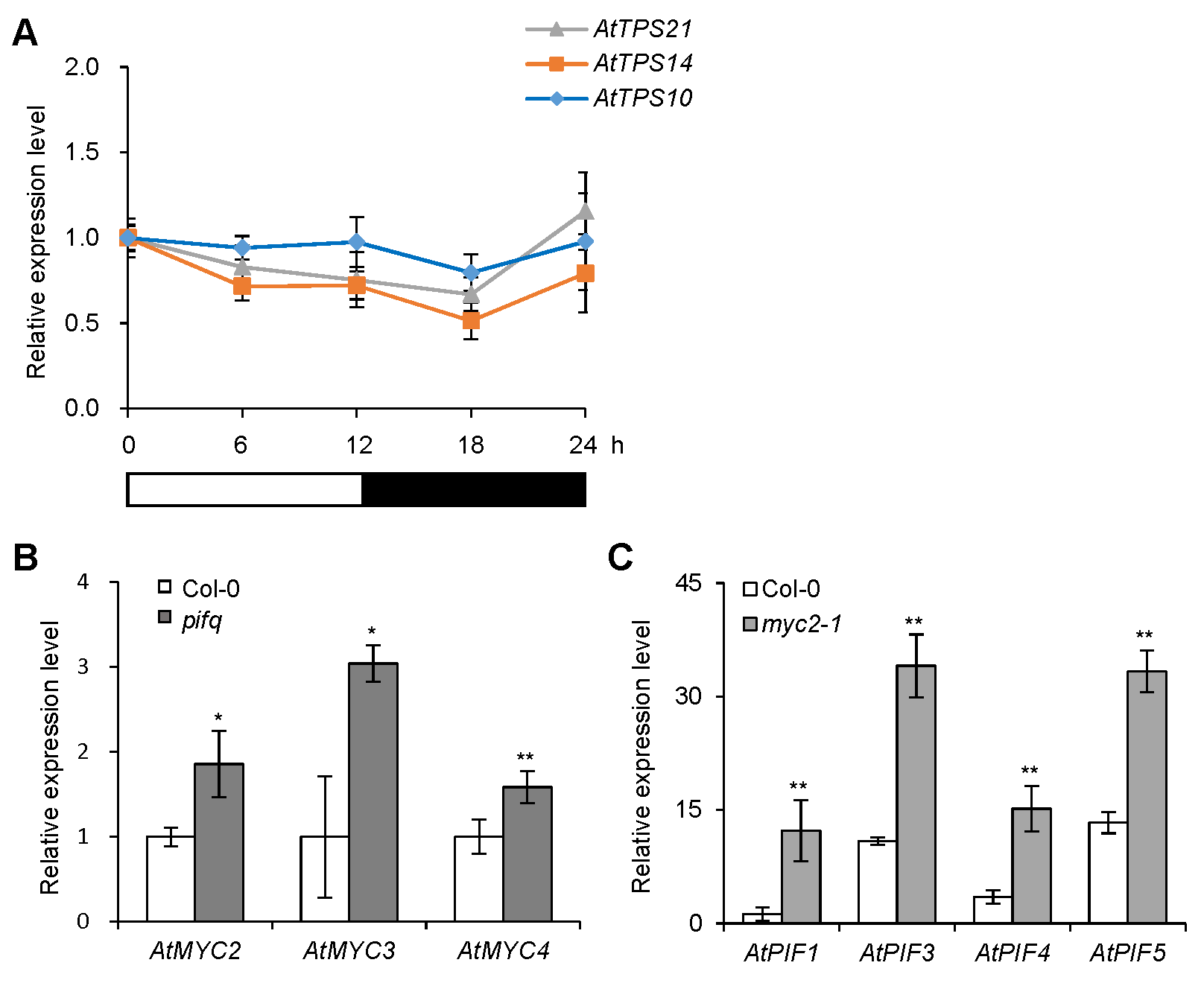

Supplement: S9 Fig — (A) The expression pattern of Arabidopsis TPS10/TPS14/TPS21 is constant during night and day time. Relative expression levels of AtTPS genes in Col-0 under 12 h light/12 h darkness. Values are mean ± SD (n = 3). (B) Relative expression levels of AtMYC genes in Col-0 and pifq mutant plants under light. (C) Relative expression levels of AtPIF genes in Col-0 and myc2-1 mutant plants under light. Values are mean ± SD (n = 3). In B-C, asterisks indicate significant differences of genes expression between Col-0 and mutant plants (*, P< 0.05; **, P< 0.01; Student’s t-test). (TIF) [file ppat.1008770.s009.tif]

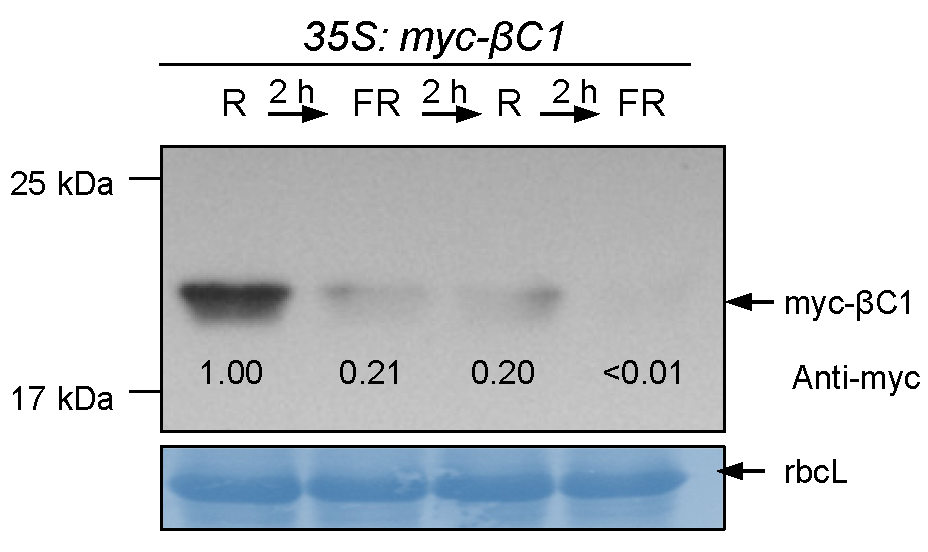

Supplement: S10 Fig — Accumulation of βC1 proteins in Nb plants after treated with continuous red light and far-red light. Plants were placed under darkness for 60 h, then transferred to continuous red light and far-red light for 2 h respectively. Samples were detected by immunoblot analysis using anti-myc antibody. Stained membrane bands of the large subunit of Rubisco (rbcL) were used as a loading control. (TIF) [file ppat.1008770.s010.tif]

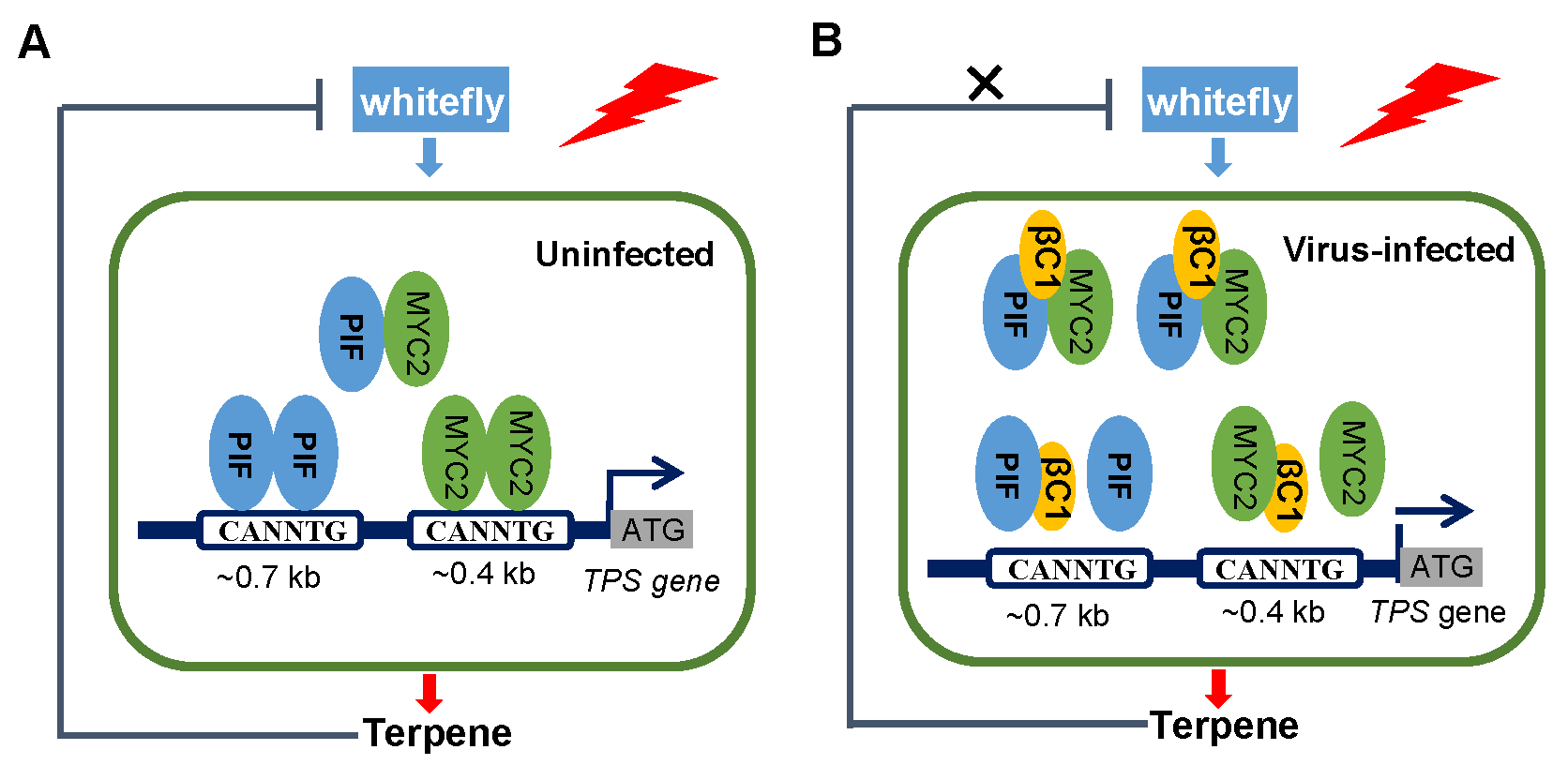

Supplement: S11 Fig — (A) In uninfected plant, both plant PIFs and MYC2 mediate the transcription of TPS genes by respectively binding to different G-box-like elements of the promoter region, and activate TPSs transcription. Thus, red-light signal and JA signal fine-tune transcription of TPS genes in plants to defend against whitefly. (B) In begomovirus-infected plants, βC1 interacts with PIFs and MYC2, and inhibits their transcriptional activity by interfering with their homodimerization and promoting AtPIFs-AtMYC2 heterodimerization. Finally, the decreased terpene synthesis and in turn enhanced whitefly performance increase the probability of pathogen transmission. (TIF) [file ppat.1008770.s011.tif]
