# Supplementary material for: Red-light is an environmental effector for mutualism between begomovirus and its vector whitefly
Source: PLoS Pathog. 2021 Jan 11;17(1):e1008770. doi: 10.1371/journal.ppat.1008770 (PMC7822537; doi:10.1371/journal.ppat.1008770)
Supplement: Attachment — Submitted filename: Review.docx [file ppat.1008770.s013.docx]

In this manuscript, the authors report red-light is an environmental effector for mutualism between begomovirus and its vector whitefly. Red-light is able to promote mutualism of whitefly-begomovirus through enhancing the stability of βC1 protein, which forms complex with PIFs. Functional analysis of PIFs indicates that PIFs positively modulate plant defense responses to whitefly via activating the transcription levels of *TSP10*. Furthermore, the exogenous MeJA assay combined with light assay confirmed that PIFs may integrates light and jasmonate signals through MYC2. Moreover, BiFC and transient assays showed that βC1 disrupts the homodimerization of PIFs and MYC2 and enhances the heterhomodimer of PIF-MYC2, therefore impairing plant defenses against begomovirus transmited by whitfly. Collectively, the authors try to demonstrate that how light signals involve in the mutualism between whitefly and its transmitted begomovirus.

The report is of interest to a broad audience and the topic is suitable for the PLoS Pathogens. The biochemical experiments were done in great detail and supported the conclusions in general. However, in my opinion, it would be more rigorous if some genetic relationships among PIFs, MYC2 and βC1 will hold up.

1) Will PIF3 overexpression be able to recover the susceptible phenotype of βC1 overexpression plants against whitefly?
2) I would like to know the phenotype of βC1 overexpression in the background of *pifq* mutant? Which will further demonstrate the relationship between βC1 and PIFs against whitefly infestation.
3) How is the phenotype of *pifq/myc2-1* feeding by whitefly? The authors demonstrated that PIFs integrates light and jasmonate signals by interaction with MYC2, and co-regulated the transcription of terpene synthase genes. However, the genetic relationship between PIFs and MYC2 is obscure.

Those experiments will clarify the relationships and strengthen the conclusions, and reveal more details between regulations of PIFs-βC1 and PIFs-MYC2.
