# Supplementary material for: Red-light is an environmental effector for mutualism between begomovirus and its vector whitefly
Source: PLoS Pathog. 2021 Jan 11;17(1):e1008770. doi: 10.1371/journal.ppat.1008770 (PMC7822537; doi:10.1371/journal.ppat.1008770)
Supplement: Attachment — Submitted filename: PLoS Pathogens-Responses final.docx [file ppat.1008770.s014.docx]

**Response to Editor’s comment**

**Q1:** As reviewers point out one of the major issues that needs to be addressed is how PIFs integrate light signaling to attract whiteflies considering PIFs are degraded in light. Since PIFs are degraded in light, how could βC1 interact with PIFs? To demonstrate that βC1 does interact with PIFs, it will be required to perform the interaction studies using commercially available PIF antibodies in light and dark conditions.

**Response:** Thanks for your critical comment. Phytochrome-PIF signaling module is evolutionarily conserved in plants and that the two gene families showed significant coevolution. PIFs act as integrators of diverse signals to modulate plant growth and development. The roles of PIFs on light condition have drawn many attentions recently. There are several evidences to show that PIFs have critical roles in plant developmental and abiotic/biotic defenses under light conditions. Beside of light signaling (including photoperiod and shade), PIFs play key roles on plant adaptations to other environmental cues such as temperature (both high and low), pathogens, internal signals such as phytohormones including auxin, brassinosteroid (BR), ethylene, gibberellic acid, and abscisic acid, the circadian clock and metabolic signals. Therefore, it is reasonable that PIFs shall function under light conditions as further explained as following reasons.

**1)** Several interacting partners involved various physiological processes have been reported for PIFs in light condition in previous studies. It was shown that DELLA proteins interact with PIF3 and PIF4 and coordinate hypocotyl elongation ([de Lucas et al., 2008](https://www.sciencedirect.com/science/article/pii/S1674205220301076?via%3Dihub#bib9); [Feng et al., 2008](https://www.sciencedirect.com/science/article/pii/S1674205220301076?via%3Dihub#bib15)). HY5 and PIF1/3 directly interact with each other and antagonistically regulate the expression of reactive oxygen species-responsive genes upon light irradiation ([Chen et al., 2013](https://www.sciencedirect.com/science/article/pii/S1674205220301076?via%3Dihub#bib7)). TIMING OF CAB EXPRESSION1 (TOC1), an essential component of the circadian clock system, interacts with the PIFs and represses their transcriptional activity, thereby regulating diverse downstream morphogenic responses ([Soy et al., 2016](https://www.sciencedirect.com/science/article/pii/S1674205220301076?via%3Dihub#bib55),). Cold-Induced CBF1 interacts and modulates PIF3 protein abundance to integrate cold response and other physiological processes (Jiang et al. 2020). All these interactions with PIFs have widely proven that PIFs play important roles for optimal plant fitness in both light and dark.

**2)** A few reports have been shown that PIF4 and PIF5 proteins accumulated to higher levels in light than in dark ([Yan](http://www.plantcell.org/content/32/7/2196.long#F9) et al. 2020; [Park et al., 2018](http://www.plantcell.org/content/32/7/2196.long#ref-61)). By contrast, PIF3 accumulated to the highest level in dark but was much less abundant in white and red light, consistent with previous reports ([Park et al., 2004](http://www.plantcell.org/content/32/7/2196.long#ref-62)).

**3)** It is true that the amount of PIF3 in light is much lower than that under dark condition. However, although phyB rapidly induces the phosphorylation and degradation of the majority of PIF3 on red light exposure, the abnormal hypocotyl phenotypes of *pif3* mutant and transgenic seedlings overexpressing PIF3 grown in red light support the conclusion that the remaining pool of PIF3 not only has a negative role on photomorphogenesis (Heng, et al. 2019) but also other roles in plant defenses in red light.

**4)** The co-presence of βC1 and PIFs on vascular tissues where the geminivirus and whitefly live with. Infected plants accumulated begomoviral βC1 proteins in the vascular tissue where they were bound to the plant vasculature-specific expression of transcription factor PIF4 (Kim et al. 2020).

**5)** We have detected the accumulation of AtPIF3 protein in light and performed Co-IP assay to prove its interaction with βC1 using endogenous AtPIF3 antibodies in light and dark conditions. Results suggested that AtPIF3 protein are indeed detectable in light (Fig. 2D. Light). Moreover, out Co-IP result showed that AtPIF3 interacts with βC1 in light and dark conditions (Fig. 2D).

**Q2:** Furthermore, as reviewers point out, a more rigorous genetic relationship between PIFs, MYC2 and βC1 should be established to demonstrate that PIFs mediate plant defense against whiteflies and βC1 inhibit PIFs and MYC2.

**Response:** Thanks for your suggestion. We have performed several genetic and molecular assays to prove the interaction of PIFs, MYC2 and βC1, beside of these published data from our and others (Li et al. 2014; Zhang et al. 2018). As a summary, we have provided several evidences to show that PIFs mediate plant defense against whiteflies. **1)** *AtPIF3*-overexpression confers enhanced *Arabidopsis* resistance to whitefly, and PIFs deficiency in *pifq* mutant promotes whitefly performance in *Arabidopsis* (Fig 3A-D); **2)** PIFs binds to the promoter of *TPS* gene and actives its expression (Fig 4A-F); **3)** the *pifq* quadruple mutants with lower expression of *TPS* genes attract more whiteflies than Col-0 plants under white or red light (Fig 3E, 3F and S5A, S5B Fig); **4)** The overexpression of *AtPIF3* or AtPIF4 can partially rescue the susceptibility of *βC1* overexpression plants against whitefly under white light (Fig 3G and 3H). **5)** In addition, during the last two months, we have generated these lines overexpressed βC1 proteins in the background of *pifq* mutant (βC1/*pifq* mutant). With these new genetic materials available, we conducted preliminary whitefly two-choice assays using *pifq* and βC1/*pifq* plants under both white light and darkness. Result showed that the additional expression of *βC1* could further increase whitefly attraction compared with *pifq* mutant in the light, but not darkness (S7C Fig.). That data are consistent with the fact that βC1 have multiple targets on JA signal pathways MYC2, AS1 and SKP1 (Li et al., 2014, Yang et al., 2008, Jia et al, 2016). Therefore, PIFs function together with MYC2 to control plant defenses against vector insects in a light-dependent mode. **6)** We conducted more genetic analysis by carrying out whitefly two-choice assay using *pifq* and *pifq/myc2-1* mutant plants in white light and darkness. Compared to *pifq* mutant, *pifq/myc2-1* mutant displayed lower defense against whitefly in light condition (As shown in Fig 6D), suggested paralleled genetic relationship for PIFs and MYC2 against whitefly.

Our data also supported our claims that geminivirus βC1 inhibit PIFs and MYC2 to achieve mutualism between whitefly and geminivirus as explained by following reasons. **1)** Our previous and new data showed that βC1 interacts with AtMYC2 and AtPIFs (Fig. 2C-E; Li et al., 2014. Fig 3). **2)** βC1 suppresses PIFs/MYC2 activity by interfering with their dimerization to inhibit plant defense against whitefly (Fig. 5; Li et al., 2014. Fig 6B-C). **3)** Whitefly prefers to βC1/*pifq* mutant than *pifq* in light (as S7C Fig. shown above), further confirming the concept that geminivirus encodes βC1 which targets multiple host proteins and achieves mutualism with whitefly.

**Reviewer #1**

**Q1: Will PIF3 overexpression be able to recover the susceptible phenotype of βC1 overexpression plants against whitefly?**

**Response:** Thanks for your good suggestion. As you suggested, if we could provide evidence to show that PIFs overexpression could be able to recover the susceptible phenotypes of βC1 overexpression plants against whitefly, then we could further prove that βC1 could target and suppress PIFs-mediated plant defense against whitefly. For this end, we performed whitefly two-choice experiments between βC1 transgenic *N.benthamiana* (βC1-1/Nb) mock inoculation plants and transiently expressed AtPIF3 or AtPIF4 on βC1-1 Nb plants. The two-choice results showed that overexpression of any of PIFs (AtPIF3 or AtPIF4) approximately decreased whitefly attraction rate to 50% of those attracted by βC1-1/Nb plants (normalized to 100%). (Fig. 3G-H). This data indicated that AtPIF3 or AtPIF4 could partially rescue the susceptible phenotype of βC1 transgenic plants against whitefly, suggesting the interaction of βC1-PIFs are functional *in vivo*

**Q2:** **I would like to know the phenotype of βC1 overexpression in the background of pifq mutant? Which will further demonstrate the relationship between βC1 and PIFs against whitefly infestation.**

**Response:** Thanks for your good suggestion. During our manuscript under 1^st^ round of reviewing, we have generated these lines overexpressed βC1 proteins in the background of *pifq* mutant (βC1/*pifq* mutant). There are very few phenotypic differences between βC1/*pifq* mutant with its parental *pifq* mutant. For example, small plant size, possible due to the additional expression of *βC1* (S7A Fig.). This phenotypic difference on possible effects on whitefly behavior needs full investigation. With these new genetic materials available, we conducted preliminary whitefly two-choice assays using *pifq* and βC1/*pifq* plants under both white light and darkness. Result showed that the additional expression of *βC1* could further increase whitefly attraction compared with *pifq* mutant in the light, but not darkness (S7C Fig.). That data are consistent with the fact that βC1 have multiple targets on JA signal pathways MYC2, AS1 and SKP1 (Li et al., 2014, Yang et al., 2008, Jia et al, 2016). In our subsequent results, we proved that AtPIFs and AtMYC2 co-regulate the expression of *AtTPS* genes and whitefly attraction in light and darkness, and βC1 suppresses AtPIFs/AtMYC2 activity by interfering with their dimerization (Fig. 5; Li et al., 2014. Fig 6B-C).

**Q3:** **How is the phenotype of pifq/myc2-1 feeding by whitefly? The authors demonstrated that PIFs integrates light and jasmonate signals by interaction with MYC2, and co-regulated the transcription of terpene synthase genes. However, the genetic relationship between PIFs and MYC2 is obscure.**

**Response:** Thanks for your comments. As you suggested, we performed whitefly two-choice experiments using Col-0 and *pifq/myc2-1* mutant plants in white light, red light and darkness. Compared to wild type Col-0 plants, *pifq/myc2-1* mutant attracted more whiteflies in white light and red light conditions (Fig. 6C), which is consist with the reduced expression of *TPSs* gene (Fig. 6B)

Next, we performed whitefly two-choice assay using *pifq* and *pifq/myc2-1* mutant plants in white light and darkness. Compared to *pifq* mutant, *pifq/myc2-1* mutant still displayed more whitefly attraction in light condition. As expected, there are no differences on whitefly attraction in darkness (Fig. 6D). As two partners of βC1, both PIFs and MYCs are members of the bHLH family. AtPIF3 and AtMYC2 bind to the different regions of *TPS* gene promoter (Fig 4A-F; Li et al., 2014. Fig 4A-B). AtMYC2 stability was increased in red light while reduced in darkness, but a reverse trend for AtPIFs stability (Chico et al., 2014; Shen et al., 2008). Additionally, the expression of AtPIFs and AtMYCs was complemented regulation in each other mutant plants (S9B-C Fig). These results suggest that AtPIFs and AtMYC2 synergistically regulate terpene biosynthesis in two paralleled pathways, and they are complemented and balanced to regulate the expression of several *TPS* genes (*TPS 10, TPS12* and *TPS21*) and whitefly attraction in white light and darkness.

**Reviewer #2**

**Q1:** **If PIFs integrates light and have the effects on whitefly attraction, there should be no difference between light and dark treatment on host attraction to whitefly when using pifq (pif1/3/4/5) quadruple mutant. How PIFs integrates light when the proteins were absent?**

**Response:** Thanks for your critical comments. This question is the same as Q1 of Editor. There are many evidences to show that PIFs have critical roles in plant developmental and abiotic/biotic defenses, please see our response to Q1 of Editor’s comment.

The preference of the whitefly for *pifq* mutant plants were compared with Col-0 plants under light and dark treatment, which could be explained by *TPSs* expression compared with Col-0 (Fig. 3F). As your suggestion, we have performed whitefly two-choice experiments between light and dark treatment in *pifq* mutant. Results show that there was no difference between light and dark treatment on host attraction to whitefly in *pifq* mutant.

It is true that the AtPIF3 accumulated to the highest level in dark but it still has less abundant in white and red light to maintain its function (Park et al., 2004). Thus, we have detected the protein accumulation of AtPIF3 in light condition and performed Co-IP assay to verify its interaction with βC1 in light and dark conditions. Results suggested that AtPIF3 protein are indeed detectable in light (Fig. 2D. Light). Our Co-IP experiment showed that AtPIF3 interacts with βC1 in light and dark conditions (Fig. 2D)

**Q2:** **In the dark, PIF proteins accumulate and directly regulate thousands of genes to maintain skotomorphogenesis. Upon illumination, the photoactived phytochromes trigger PIFs’ rapid phosphorylation and subsequently proteasome-mediated degradation. In the dark, PIF proteins accumulated, and if PIFs regulate the transcription of terpene synthase genes, the expression of AtTPS10、AtTPS14 and AtTPS21 should be upregulated. But they were not (Figure 4E). In the light, PIF proteins were degraded, and if PIFs regulate the transcription of terpene synthase genes, the expression of AtTPS10、AtTPS14 and AtTPS21 should be downregulated in the wild type Col 0, but they were not, either (Figure 4E-G).**

**Response:** Thanks for your comments. In the new version of manuscript, we have provided data that AtPIFs and AtMYC2 integrated regulated *TPSs* expression to involve in whitefly host preference. AtPIF3 or AtMYC2 binds to the different regions of *AtTPS* gene promoter (Fig 4A-F; Li et al., 2014. Fig 4A-B). However, AtPIF3 binds to the distal end of the *AtTPS* gene promoter (Fig 4A-F), and AtMYC2 binds to the proximal end of the *AtTPS* gene promoter (Li et al., 2014. Fig 4A-B), thus AtMYC2 was more transcriptionally active than AtPIFs (Fig 6E). Previous studies demonstrated that AtMYC2 stability was reduced in darkness while increased in red light, but a reverse trend for AtPIFs stability (Chico et al., 2014; Shen et al., 2008). In light, PIFs level was deceased, but AtMYC2 level was increased, and AtMYC2 was more transcriptionally active than PIFs, so the *AtTPS* genes expression was upregulated in Col-0 plants in the light. Vice versa, the *AtTPS* genes expression was downregulated in Col-0 plants in darkness.

**Q3:** **If PIFs regulate the transcription of terpene synthase genes and the expression of terpene synthase genes is light-dependent, the expression of AtTPS10、AtTPS14 and AtTPS21 should have no difference between light and dark treatment when using pifq (pif1/3/4/5) quadruple mutant in the dark. When PIFs were disrupted, How the mutants could still respond to light?**

**Response:** Thanks for your comments. As our above data suggested, the transcription of *AtTPS* genes was co-regulated by AtPIFs and AtMYC2. Both AtPIF3 and AtMYC2 bind to different G-box-like elements of the *AtTPSs* promoter region, and activate its transcription (Fig 4A-F). Previous study has proved that JA and GA also co-regulate *TPSs* expression (Bottom figure A) (Hong et al., 2012), thus at least light, jasmonate (JA) and gibberellin (GA) three pathways involved in the regulation of *TPSs* gene expression. So we speculate that AtPIF-mediated *AtTPSs* expression might be also regulated by GA. In the white/red light, JA signaling is activated in wild-type Col-0 plants. The activated JA signaling promotes the degradation of JAZ9, releasing RGA (a DELLA repressor protein) from JAZ9-RGA complex, which in turn forms another PIF3-RGA complex to inactivate PIF3 activity (Bottom figure B) (Paik et al., 2017; Yang et al., 2012). However, in *pifq* mutant, because of the absent of PIF3 proteins, the released-RGA will suppress GA signaling, resulting in inhibition of *TPSs* expression. Under dark/far-red light, it is unable to activate JA signaling, so it could not trigger subsequently processes. Therefore, *pifq* mutant plants are more attractive than Col-0 plant to whiteflies under white or red light, but not under dark, far-red or blue light.

**(A)** GA induced *TPSs* expression (Hong et al., 2012). **(B)** Model of JA Regulate growth–defense tradeoffs in Arabidopsis (Paik et al., 2017).

**Q4:** **In the light, PIF proteins were degraded. The host attraction to whitefly is caused by white or red light. If the light effect is due to the interaction of βC1 with PIFs. How βC1 could interact with the PIF proteins that are not accumulated at all during the day light time.**

**Response:** Thanks for your critical comment. It is true that the AtPIF3 accumulated to the highest level in dark but it still has less abundant in white and red light to maintain its function (Park et al., 2004). We have performed Co-IP assay to verify its interaction with βC1 in light and dark conditions. Results suggested that AtPIF3 protein are detectable in light (Fig. 2D. Light), and AtPIF3 interacts with βC1 in light and dark conditions (Fig. 2D)

**Q5:** **In Fig 3A, we cannot see the results. This figure was not displayed normally.**

**Response:** We regret the mistake in Fig 3A in the last version. In the new revision, we have double-checked and confirmed that all figures were displayed normally.

**Q6:** **Using BiFC assays to show the interaction between AtMYC2 with AtPIF3 and AtPIF4 (S6 Fig) are misleading, BiFC assays are easy to cause false positive results. The authors should provide the yeast two hybrid assay to confirm their interactions.**

**Response:** Thanks for the comments. In BiFC assay, we have used empty vector nYFP as negative control to exclude false positive results. In addition, Zhang et al. (2018) have proved that AtPIF1/3/4/5 interacts with AtMYC2 using luciferase complementation assays and further confirmed the interaction between AtPIF4 and AtMYC2 by pull-down assay (Zhang et al., 2018. Fig 6A and 6C), which could double confirm our interaction results for AtMYC2 with AtPIF3 and AtPIF4.

Luciferase complementation assays and pull-down assay proved the interaction between AtPIFs and AtMYC2 (Zhang et al., 2018)

**Q7:** **The finding that βC1 accumulated in light but not in dark is interesting. The light effect on host attraction to whitefly may due to other mechanism but not interference of PIFs pathway by βC1.**

**Response:** Thanks for your suggestion. Similar with our response to Q2 of Editor, Our data also supported our claims that geminivirus βC1 inhibit AtPIFs and AtMYC2 to achieve mutualism between whitefly and geminivirus. **(1)** PIFs indeed could bind the promotor of *TPSs* genes and regulate its expression in a light-dependent manner (Fig. 4); **(2)** AtPIFs mediate plant defense against whitefly in Arabidopsis (Fig. 3A-D); **(3)** βC1 protein interacts with AtPIF3 proteins in light and dark conditions (Fig. 2D); **(4)** βC1 suppresses AtPIFs activity by interfering with its dimerization (Fig. 5). These results demonstrated that PIFs indeed participate in plant defense against whitefly, interference of PIFs pathway by βC1 is one mechanism that the light effect on host attraction to whitefly.

Meanwhile, Fig 2A showed that the accumulation of βC1 protein was higher in red light than that in far-red light Interestingly, βC1 protein accumulation was reduced again when treated with an interval of far-red light (S10 Fig), indicating that the photoreceptors phyB or phyA might target viral βC1 protein to regulated its stability and plant defense against whitefly, which invites our further detail investigation in the future. We regard this is reasonable that βC1 might interfere with plant light signaling via targeting more than PIFs since it is been aimed by PIFs in turn.

**Q8:** **In Fig 5H, I strongly recommended to include a schematic diagram showing constructs for the effectors and the AtTPS10 Promoter: LUC reporter.**

**Response:** Thanks, we have added the schematic diagram (Fig 4G).

**Q9:** **In abstract，Page 6，line 37 “inhibit” should be changed to “inhibits”.**

**Response:** Thanks, we have changed the word.

**Q10: In introduction，Page 6，line 50 “affect” should be changed to “affects”.**

**Response:** Thanks, we have changed the word.

**Q11:** **Fig 1D “β C1” should be changed to “βC1”.**

**Response:** Thanks, we have changed it.

**Q12:** **In reference，Page 29，line 558 “Interactions” should be changed “interactions”; Page 32，line 676 “Interacting Factors” should be changed “interacting factors”.**

**Response:** Thanks, we have changed the words.

**Reviewer #3**

**Q1:** **In Fig 2 and S9, a control foreign protein should be detected to show that βC1 was specifically decreased in darkness. Also, surprisingly, fluorescence from YFP-fused βC1 in darkness was at a similar level to that in white light (S3 Fig). How to explain this?**

**Response:** Thanks for your suggestion, we have detected the protein accumulation of a control foreign protein YFP under different light treatments (S3C Fig). The results show that light could not affect the accumulation of YFP protein under different light treatments.

In the last manuscript, the fluorescence from YFP-fused βC1 was shown in a single cell to display the subcellular localization of βC1 protein. In the new version, we have provided a broader perspective to observe the fluorescence accumulation of YFP-fused βC1 protein (S3A Fig).

**Q2:** **In Fig 4, AtPIF3-OE was used for analysis. If βC1 was expressed in AtPIF3-OE, could the βC1-mediated whitefly preference be impaired?**

**Response:** Thanks for your suggestion. We have performed whitefly two-choice experiments using βC1-1/Nb mock inoculation plants and AtPIF3 or AtPIF4 transiently expressed on βC1-1 plants. Results showed that compared with βC1-1/Nb plants (normalized to 100%), overexpression of any of PIFs (AtPIF3 or AtPIF4) approximately decreased whitefly attraction rate to 50% (Fig. 3G-H), which indicated that AtPIF3 or AtPIF4 overexpression in βC1-1/Nb plants could partially rescue the susceptible phenotype of βC1 transgenic plants against whitefly, suggesting the interaction of βC1-PIFs are functional *in vivo*

**Q3:** **In Fig 6C, βC1 increased the interaction signal of AtPIF4-cEYFP and nEYFP-AtMYC2. How do you exclude the roles of βC1 as suppressor of RNA silencing that could increase the expression level of both proteins? Moreover, in Fig 6E, with the increase of His-βC1, the amount of pulled down GST-AtMYC2 was not changed accordingly. Does it mean that βC1 only function as VSR improving the level of foreign protein?**

**Response:** Zhong et al (2017) have reported that mimic phosphorylation of Ser-33 of the βC1 protein (serine to aspartate, βC1^S33D^) impairs its functions as a viral suppressor of RNA silencing, so we use βC1^S33D^ mutant as negative control to perform competitive BiFC experiments. There are no significant differences between βC1 protein and βC1^S33D^ protein in effects on the interaction signal of AtPIF4-cEYFP and nEYFP-AtMYC2 (Fig. 7C-7D). The results can exclude the roles of βC1 as viral suppressor of RNA silencing to improve the expression level of foreign protein.

**Q4:** **Results in this paper demonstrate the function of PIFs in the interaction among begomovirus, vector and plant, while the mechanism of red-light to stabilize βC1 is not clarified clearly. I suggest authors reorganizing the paper to focus on function of PIFs and its relationship to MYC2.**

**Response:** Thanks for your suggestion. In the new revision, we have reorganized the paper to focus on function of PIFs and its relationship to MYC2.

**(1)** For the function of PIFs: we proved that AtPIFs bind to the promotor of *TPSs* genes and regulate its expression in a light-dependent manner (Fig. 4A-F), and mediated plant defense to whitefly (Fig. 3A-D).

**(2)** For the relationship between AtPIFs and AtMYC2**:** our previous and new results showed that AtPIF3 and AtMYC2 binds to the different regions of *TPS* gene promoter (Fig 4A-F; Li et al., 2014. Fig 4A-B). Additionally, the expression of AtPIFs and AtMYC2 was complemented regulation in each other mutant plants (S9B-C Fig). AtMYC2 stability was increased in red light while reduced in darkness, but a reverse trend for AtPIFs stability (Chico et al., 2014; Shen et al., 2008). These results suggest that AtPIFs and AtMYC2 synergistically regulate terpene biosynthesis in two paralleled pathways, and they are balanced to regulate the expression of *AtTPS* genes and whitefly attraction in white light and darkness.

**(3)** For βC1 protein: previous result suggested that βC1 interacts with AtMYC2 (Li et al., 2014. Fig 3). Our new data proved that βC1 interacts with AtPIF3 proteins in light and dark conditions (Fig. 2D). βC1 suppresses AtPIFs/AtMYC2 activity by interfering with their dimerization (Fig. 5; Li et al., 2014. Fig 6B-C).

**Q5:** **Line 86. Actually, the mechanism of how light affects this interaction is not clarified here either. It’s better to modify this sentence.**

**Response:** Thanks, we have modified this sentence.

**Q6:** **Line 90. Virus name here should not be italic. Please see https://talk.ictvonline.org/information/w/faq/386/how-to-write-virus-and-species-names**

**Response:** Thanks, we have modified the state.

**Q7:** **Line 112 and 124. “Nb” should be italic? Please check the full text.**

**Response:** Thanks, Nb cannot be italic. We have checked the full text.

**Q8:** **In Fig 5A, do promoters of TPS14 and 21 have similar elements to that of TPS10? Are they also regulated by PIFs?**

**Response:** The promoters of *TPS14* and *TPS21* also have G-box like elements, which are similar with that of *TPS10*. We performed ChIP-PCR and confirmed that AtPIF3 also directly bind to their promoters, suggesting that *TPS14* and *TPS21* are also regulated by AtPIF3 (Fig. 4C-F).

**Q9:** **In Fig 5C, total YFP-fused proteins should be detected to ensure that proteins in GUS and βC1 treatment were at a similar level.**

**Response:** Thanks for the comments. As you suggested, we have performed western blot to detect the protein expression in BiFC assay. Since our YFP antibody was unable to recognize the N- terminal of YFP proteins, we detect the accumulation of AtPIF3-cYFP in normal light. The results show that total YFP-fused proteins in GUS and βC1 treatment were at a similar level (S5C Fig).

**Q10:** **In Fig 6E, please provide additional evidences to confirm three proteins are in a complex. Also, it had better determine the responsible domains of βC1, PIF and MYC2 for interaction, which would help understand their interaction.**

**Response:** Thanks for your suggestions. We have performed Co-IP assay to confirm βC1, AtPIF3 and AtMYC2 are in a complex. We used YFP-bead to enrich YFP-βC1 protein, western blot result suggested that YFP-βC1 is able to enrich AtPIF3-HA and Myc-AtMYC2 protein (Fig. 6A). In addition, Li et al (2014) have reported that βC1 binds to the bHLH domain of the AtMYC2 protein (Li et al., 2014. Fig 6A). In this manuscript, we also apply data that βC1 interacts with the bHLH domain of AtPIF3 (2E Fig). These results indicated that βC1 function as a bridge to promote the interaction between AtPIF3 and AtMYC2.

**Reference**

Chen D, Xu G, Tang W, Jing Y, Ji Q, Fei Z, Lin R. 2013.Antagonistic basic helix-loop-helix/bZIP transcription factors form transcriptional modules that integrate light and reactive oxygen species signaling in Arabidopsis. The Plant Cell. 25: 1657–1673. DOI: 10.1105/tpc.112.104869. PMID: 23645630.

Chico, J. M., Fernández-Barbero, G., Chini, A., Fernández-Calvo, P., Díez-Díaz, M., Solano, R. 2014. Repression of jasmonate-dependent defenses by shade involves differential regulation of protein stability of MYC transcription factors and their JAZ repressors in Arabidopsis. The Plant Cell **26**:1967-1980. DOI:10.1105/tpc.114.125047, PMID: 24824488.

de Lucas M, Davière J.M, Rodríguez-Falcón M, Pontin M, Iglesias-Pedraz J.M, Lorrain S, Fankhauser C, Blázquez M.A, Titarenko E, Prat S. 2008. A molecular framework for light and gibberellin control of cell elongation. Nature 451, 480–484. DOI: 10.1038/nature06520. PMID: 18216857.

Feng S, Martinez C, Gusmaroli G, Wang Y, Zhou J, Wang F, Chen L, Yu L, Iglesias-Pedraz J.M, Kircher S, Schäfer E, Fu X, Fan L.M, Deng X.W. 2008. Coordinated regulation of Arabidopsis thaliana development by light and gibberellins. Nature. 451, 475-479. DOI: 10.1038/nature06448. PMID: 18216856.

Heng Y, Jiang Y, Zhao X, Zhou H, Wang X, Deng X.W, Xu D. 2019. BBX4, a phyB-interacting and modulated regulator, directly interacts with PIF3 to fine tune red light-mediated photomorphogenesis. Proc Natl Acad Sci USA. 116, 26049-26056. DOI: 10.1073/pnas.1915149116. PMID: 31776262.

Hong, G. J., Xue, X. Y., Mao, Y. B., Wang, L. J., Chen, X. Y. 2012. Arabidopsis MYC2 interacts with DELLA proteins in regulating sesquiterpene synthase gene expression. The Plant Cell **24**, 2635-2648. DOI: 10.1105/tpc.112.098749, PMID: 22669881.

Jia Q, Liu N, Xie K, Dai Y, Han S, Zhao X, Qian L, Wang Y, Zhao J, Gorovits R, Xie D, Hong Y, Liu Y. 2016. CLCuMuB βC1 Subverts Ubiquitination by Interacting with NbSKP1s to Enhance Geminivirus Infection in *Nicotiana benthamiana*. PLoS Pathog. **12**(6):e1005668. DOI: 10.1371/journal.ppat.1005668. PMID: 27315204.

Jiang B, Shi Y, Peng Y, Jia Y, Yan Y, Dong X, Li H, Dong J, Li J, Gong Z, Thomashow M.F, Yang S. 2020. Cold-Induced CBF-PIF3 Interaction Enhances Freezing Tolerance by Stabilizing the phyB Thermosensor in Arabidopsis. Mol Plant. 13, 894-906. DOI: 10.1016/j.molp.2020.04.006. PMID: 32311530.

Kim S, Hwang G, Kim S, Thi T.N, Kim H, Jeong J, Kim J, Kim J, Choi G, Oh E. 2020. The epidermis coordinates thermoresponsive growth through the phyB-PIF4-auxin pathway. Nat Commun. 11,1053. DOI: 10.1038/s41467-020-14905-w. PMID: 32103019.

Li, R., Weldegergis, B. T., Li, J., Jung, C., Qu, J., Sun, Y., Qian, H. Tee, C., van Loon, J. J., Dicke, M. 2014. Virulence factors of geminivirus interact with MYC2 to subvert plant resistance and promote vector performance. The Plant Cell **26**:4991-5008. DOI:10.1105/tpc.114.133181, PMID: 25490915.

Park E, Kim J, Lee Y, Shin J, Oh E, Chung WI, Liu J.R, Choi G. 2004. Degradation of phytochrome interacting factor 3 in phytochrome-mediated light signaling. The Plant Cell Physiol. 45,968-975. DOI: 10.1093/pcp/pch125. PMID: 15356322.

Park E, Kim Y, Choi G. 2018. Phytochrome B Requires PIF Degradation and Sequestration to Induce Light Responses across a Wide Range of Light Conditions. The Plant Cell. 30, 1277-1292. DOI: 10.1105/tpc.17.00913. PMID: 29764986.

Paik, I., Kathare, P. K., Kim, J. I., Huq, E. 2017. Expanding roles of PIFs in signal integration from multiple processes. Mol Plant. **10**:1035-1046. DOI: 10.1016/j.molp.2017.07.002, PMID: 28711729.

Shen, H., Zhu, L., Castillon, A., Majee, M., Downie, B., & Huq, E. 2008. Light-induced phosphorylation and degradation of the negative regulator PHYTOCHROME-INTERACTING FACTOR1 from Arabidopsis depend upon its direct physical interactions with photoactivated phytochromes. The Plant Cell **20**:1586-1602. DOI:10.1105/tpc.108.060020, PMID: 18539749.

Soy J, Leivar P, González-Schain N, Martín G, Diaz C, Sentandreu M, Al-Sady B, Quail P.H, Monte E. 2016. Molecular convergence of clock and photosensory pathways through PIF3-TOC1 interaction and co-occupancy of target promoters. Proc Natl Acad Sci USA. 113, 4870-4875. DOI: 10.1073/pnas.1603745113. PMID: 27071129.

Yan Y, Li C, Dong X, Li H, Zhang D, Zhou Y, Jiang B, Peng J, Qin X, Cheng J, Wang X, Song P, Qi L, Zheng Y, Li B, Terzaghi W, Yang S, Guo Y, Li J. 2020. MYB30 Is a Key Negative Regulator of Arabidopsis Photomorphogenic Development That Promotes PIF4 and PIF5 Protein Accumulation in the Light. The Plant Cell. 32, 2196-2215. DOI: 10.1105/tpc.19.00645. PMID: 32371543.

Yang, D. L., Yao, J., Mei, C. S., Tong, X. H., Zeng, L. J., Li, Q., Xiao L.T., Sun T. P., Li J., Deng X. W., Lee C. M., Thomashow M. F., Yang Y., He Z., He, S. Y. 2012. Plant hormone jasmonate prioritizes defense over growth by interfering with gibberellin signaling cascade. Proc Natl Acad Sci USA **109**: E1192-E1200. DOI: 10.1073/pnas.1201616109, PMID: 22529386.

Yang J.Y, Iwasaki M, Machida C, Machida Y, Zhou X, Chua NH. 2008. βC1, the pathogenicity factor of TYLCCNV, interacts with AS1 to alter leaf development and suppress selective jasmonic acid responses. Genes Dev. **22**:2564-2577. Doi.org/10.1101/gad.1682208. PMID: 18794352.

Zhang, X., Ji, Y., Xue, C., Ma, H., Xi, Y., Huang, P., Wang H., An F., Li B., Wang Y., Guo, H. 2018. Integrated regulation of apical hook development by transcriptional coupling of EIN3/EIL1 and PIFs in Arabidopsis. The Plant Cell **30**:1971-1988. DOI: 10.1105/tpc.18.00018, PMID: 30104405.

Zhong X, Wang Z.Q, Xiao R, Cao L, Wang Y, Xie Y, Zhou X. 2017. Mimic phosphorylation of a βC1 protein encoded by TYLCCNB impairs its functions as a viral suppressor of RNA silencing and a symptom determinant. J Virol. **91**:e00300-e00317. DOI: 10.1128/JVI.00300-17. PMID: 28539450.
